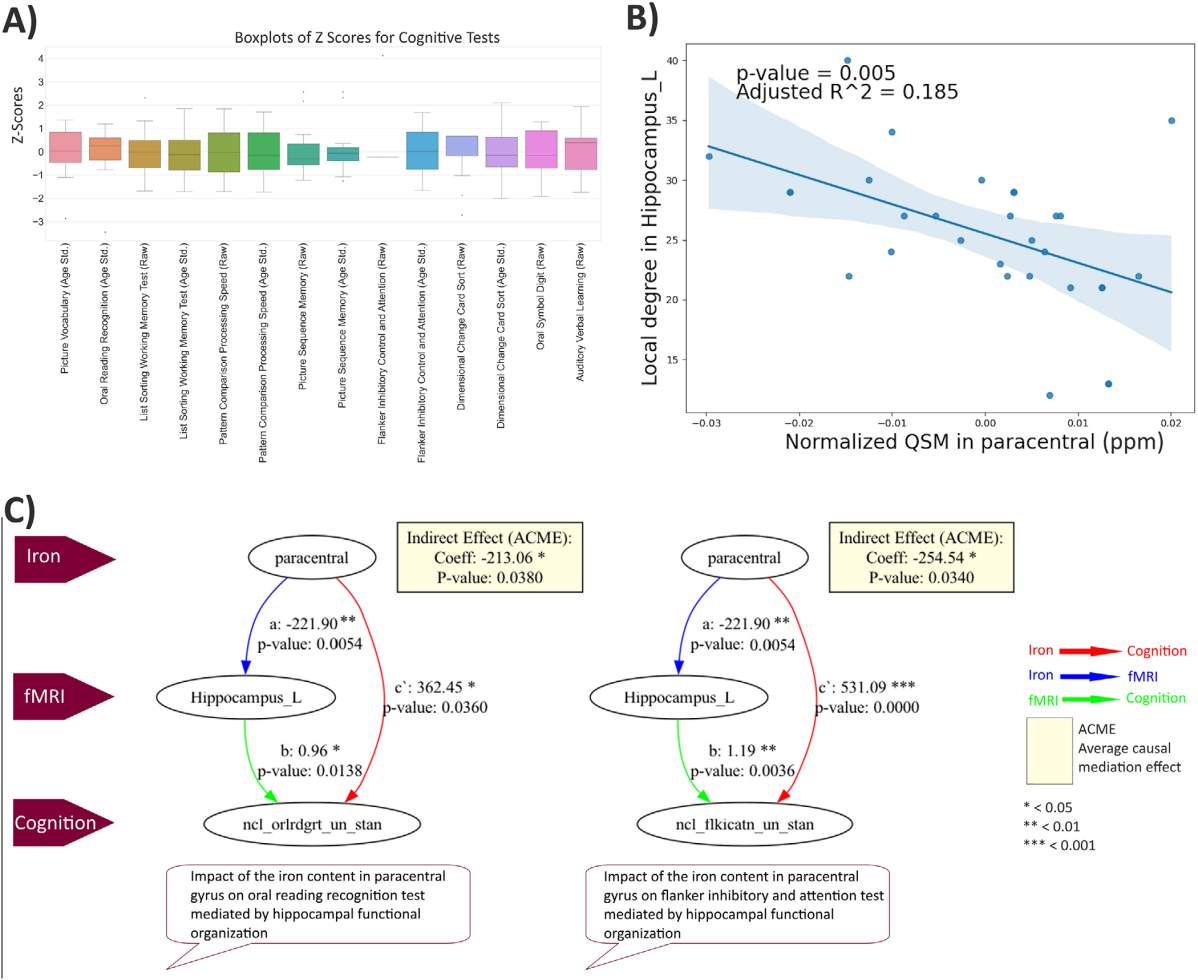# Hippocampal Functional Organization Mediates the Impact of the Cortical Iron on the Cognition in Women with Suspected Coronary Microvascular Dysfunction

**DOI:** 10.1002/alz.093612

**Published:** 2025-01-09

**Authors:** Arzu C Has Silemek, Jeffrey C Wertheimer, Janet Wei, Oana M Dumitrascu, Sarah Kremen, Yibin Xie, Debiao Li, Michael D Nelson, Zaldy S Tan, Noel Bairey Merz, Pascal Sati, Wei Gao

**Affiliations:** ^1^ Cedars‐Sinai Medical Center, Los Angeles, CA USA; ^2^ Barbra Streisand Women’s Heart Center, Smidt Heart Institute, Cedars‐Sinai Medical Center, Los Angeles, CA USA; ^3^ Mayo Clinic College of Medicine and Science, Scottsdale, AZ USA; ^4^ The University of Texas at Arlington, Arlington, TX USA

## Abstract

**Background:**

Women with suspected coronary microvascular dysfunction (CMD) may be at higher risk of experiencing cognitive decline due to cerebral small vessel disease, a known contributor to Alzheimer’s disease and related dementias (ADRD). A potential underlying mechanism that could accelerate this cognitive decline is the accumulation of brain tissue iron, which has been previously linked to changes in brain function potentially caused by oxidative stress and cell death. Therefore, we aim to elucidate whether a similar mechanism could affect women with suspected CMD by investigating the potential role of iron deposition on the brain’s functional organization and its effect on cognition using advanced magnetic resonance imaging (MRI).

**Material and Methods:**

Twenty‐seven women with suspected CMD [Age; median (range) = 54 (29‐76)], drawn from ongoing cohorts (3R01HL146158‐04S1,3U54AG065141‐04S1), underwent a 3T MRI protocol, including submillimeter T2* 3‐dimensional echo‐planar‐imaging for assessing iron deposition with high‐resolution quantitative susceptibility mapping (QSM) and resting‐state fMRI (rs‐fMRI). Iron content was quantified by total‐generalized‐variation based QSM analysis. Functional integrity was determined via graph theoretical approach (i.e., nodal degree). Cognitive assessment was also performed using the NIH Toolbox. Mediation analysis was conducted using Python Statsmodels.

**Results:**

Most of the women with suspected CMD showed lower performance in processing speed, working memory, executive function, and attention (Figure‐1A). We found a significant association between elevated iron levels in paracentral gyrus and lower functional connectivity in left hippocampus (p = 0.005, adjusted‐r2 = 0.19) (Figure‐1B). Elevated iron level in the paracentral gyrus showed an impact on cognitive performance in the domains of executive function and attention (p<0.0001, coefficient = 531.09) as well as language functions and crystalized abilities (p = 0.036, coefficient = 362.45), mediated by functional connectivity in the left hippocampus (indirect effect on executive function / language: p = 0.034 / 0.038, coefficient = ‐254.54 /‐ 213.06) (Figure‐1C).

**Conclusions:**

Our results suggest that changes in hippocampal functional organization are associated with cortical iron deposition and mediate its impact on cognitive performances. These changes may increase the risk of cognitive decline/ADRD or in women with suspected CMD. Future research in a larger cohort with a longitudinal design is necessary to validate and expand upon these findings.